# Balancing Nutritional and Environmental Sustainability Through the Evaluation of the Water Footprint of the Recommended Italian, Spanish, and American Diets

**DOI:** 10.3390/nu17010023

**Published:** 2024-12-25

**Authors:** Giulia Camporesi, Alessandra Bordoni

**Affiliations:** 1 Department of Agricultural and Food Sciences (DISTAL), University of Bologna, Piazza Goidanich 60, 47521 Cesena, FC, Italy; giulia.camporesi9@unibo.it; 2 Interdepartmental Centre for Industrial Agri-Food Research (CIRI), University of Bologna, Via Quinto Bucci 336, 47521 Cesena, FC, Italy

**Keywords:** water footprint, sustainability, food-based dietary guidelines, Italian Dietary Guidelines, Spanish Dietary Guidelines, Dietary Guidelines for Americans, food choice

## Abstract

**Background/Objectives**: The water footprint (WF) provides information on the impact of individual foods on water consumption, but to better direct food production toward water saving, we need to understand how to reduce the WF of our diets while keeping it healthy. In this study, we compared the WF of healthy diets based on national food-based dietary guidelines with the aim of highlighting changes in dietary patterns that could reduce water requirements without compromising nutritional adequacy. **Methods**: Three 2000 kcal/day dietary patterns were elaborated following the Italian, Spanish, and American dietary guidelines, and their total, green, blue, and grey WFs were calculated. **Results**: The Italian dietary pattern showed the lowest total WF (2806 L per capita/day), with the American and Spanish patterns being 8% and 10.5% higher, respectively. The food groups contributed differently to the total WF. In the USA, animal foods were the main contributor (56% versus 41% in Spain and 38% in Italy). The contribution of plant foods was higher in Italy (61%) than in Spain (54%) and the USA (38%). The distribution of the total WF between WF_green_, WF_blue_, and WF_grey_ was similar across the dietary patterns. Within each food group, and mainly in the animal-origin food group, the type of product significantly modulated the WF. **Conclusions**: Different diets can be equally nutritionally sustainable but have different impacts on environmental sustainability. The comparison of their WFs can be the starting point to promote dialogue between nutritionists, operators in the environmental sector, and the agri-food industry to ensure a healthy and balanced approach.

## 1. Introduction

In recent decades, the concern for water scarcity has increased, as two-thirds of the world’s population experience severe water scarcity during at least part of the year [1]. This concern is growing as the water demand is expected to increase in the future [2] due to further population growth, improving living standards, and changes in consumption patterns [2]. Indeed, the global food demand is expected to increase by 35% to 56% between 2012 and 2050 [3], and this will consequentially lead to a higher environmental impact of dietary habits. Previous works have investigated the environmental impact of different diets (omnivore, lacto-ovo-vegetarian, vegan, etc.), focusing in particular on greenhouse gas emissions and on how to reduce the carbon footprint and the impact on climate change [4]. However, as we are already experiencing climate change effects, it is also important to evaluate their consequences on food production and how to manage the needed resources, particularly water withdrawals. Climate change has a growing impact on water scarcity and exacerbates the temporal and geographical mismatch between water demand and its availability [5]. 

As agriculture is responsible for 70% of total water withdrawal [6], our knowledge of the water needs for food security and a healthy and high-quality diet is the starting point for making further decisions about food production worldwide. The sum of the water needed in the process of food production, measured over the full supply chain, is defined as the water footprint (WF) [7] and is expressed in water volume per unit of product (usually m3 ton−1). Different water sources contribute to the total WF: (i) green WF (WF_green_) represents rainfalls; (ii) blue WF (WF_blue_) is the consumption of surface and groundwater resources, which include water evaporation and incorporation into a product; and (iii) grey WF (WF_grey_) is defined as the freshwater needed to assimilate pollutants. 

WF provides direct information about the impact of single food items on water consumption, but to suggest more sustainable dietary patterns, further data elaboration is needed. Sustainable diets are those with low environmental impacts that contribute to food and nutrition security and healthy living for present and future generations. Sustainable diets are protective and respectful of biodiversity and ecosystems, culturally acceptable, accessible, economically fair and affordable, nutritionally adequate, safe, and healthy while optimizing natural and human resources [8]. 

The nutritional adequacy and health perspective of sustainability of diets is the focus of the food-based dietary guidelines (FBDGs). Most countries have national FBDGs that provide recommendations for a healthy diet; they are based on the nutrient reference intakes that are similar between countries as they are the result of scientific knowledge in health and nutrition. Although based on the same principles, national FBDGs reflect the country’s habits and traditions in the matter of the consumption of different foods, and this could have an impact on the environmental footprint of the diet. In fact, since foods are complex matrices, many different combinations may result in a similarly correct energy and nutrient intake, but not in the same environmental impact.

In a previous study [9], the total WF of the dietary healthy patterns suggested by the Italian FBDGs (IDGs) [10] was calculated based on the information reported in Mekonnen et al. [11,12]. The objective of the present study is to increase our knowledge of the water requirements for a healthy diet and understand if it differs between countries due to dietary habits. To this purpose, we evaluated and compared the WFs of three dietary patterns recommended by national FBDGs: Italian; Spanish, representative of the Mediterranean diet; and the USA, representative of a Western diet. The final aim was to highlight possible modifications in dietary patterns that could decrease the WF without affecting their nutritional adequacy.

The information provided by the Dietary Guidelines for Americans (DGAs) [13] and the Spanish Dietary Guidelines (SDGs) [14] were used to elaborate on 2000kcal/day dietary healthy patterns, and the 2000 kcal/day dietary pattern already considered in Bordoni A. [9] was used to represent the Italian healthy diet. The total, green, blue, and grey WFs were then calculated based on the information reported by Mekonnen et al. [11,12]. 

## 2. Methods

Three dietary patterns providing 2000 kcal/day were elaborated following the suggestion of the IDGs, DGAs, and SDGs. The WF per kg (WF/kg) of the food products listed in each dietary pattern was calculated using the data reported by Mekonnen and Hoekstra [11,12] and considering the green, blue, and grey WF. For animal products, the global average and the weighted average of the farming system were considered. To reflect national preferences as closely as possible, in cases where the guidelines did not specify exactly which items were included in a food group, we used national consumption data, choosing the most consumed products in the group to calculate the WF.

The weekly WF (WF/week) of the three dietary patterns was calculated by multiplying the WF/kg of each food category by the suggested serving size and weekly consumption and summing up all results. As in the Dietary Guidelines, the quantity of food that corresponds to a serving is expressed as the weight of the edible part, while the WF is calculated as the production weight, so a conversion was made where necessary (e.g., fruits) based on the data reported in CREA [15]. 

The details are as follows:

### 2.1. Italy

The medium-energy dietary pattern (2000 kcal/day) suggested by the IDGs was considered. In the IDGs, the serving sizes and the suggested number of daily/weekly servings that should be consumed by Italian healthy adults are reported for the different food categories. The calculation of the green, blue, and grey WFs of the diet was performed as reported in Bordoni A. [9], except for the seafood group. In this work, the WF of seafood was calculated using the information provided by Pahlow et al. [16] since the reference used in the previous work did not discriminate between blue, green, and grey WFs.

### 2.2. The USA

To compile a 2000 kcal/day dietary pattern corresponding to a balanced diet for healthy American adults, the DGA recommendations on serving size and frequency of consumption of the different food groups were followed. The serving sizes reported in cup equivalents were converted into grams using the information about serving size provided by USDA in the Food and Nutrient Database for Dietary Studies (FNDDS) [17]. As the DGAs suggest different items within each food group (e.g., “Fruits. All fresh, frozen, canned, and dried fruits and 100% fruit juices), at least four suggested foods for each group were considered, selecting the mostly consumed foods in the US based on the American consumption habits [18]. The WFs of the recommended servings of the selected foods in the group were averaged and the resulting WFs used for calculation.

Food group 1 (Vegetables). In the DGAs, this group is divided into five subgroups, and for each subgroup, a weekly frequency of consumption is suggested. In each subgroup, we selected the foods listed in the DGAs example lists that were reported as the most consumed vegetables in the USA [18]: (i) dark-green vegetables: lettuce, broccoli, kale, and spinach; (ii) red and orange vegetables: tomatoes, squash, sweet potatoes, carrot, and bell pepper; (iii) beans, peas, and lentils: dry beans, peas, lentils, and chickpeas; (iv) starchy vegetables: corn, plantain, lima beans, and white potatoes; and (v) other vegetables: cabbage, avocado, turnip, onion, eggplant, and cucumber. For legumes, since the WF is reported for dried legumes and the recommended serving size is one-quarter cup of cooked legumes, a conversion factor of 2.5 was applied. 

Food group 2 (Fruits). The WF was calculated as the average of the WF of the fifteen most consumed fruits in the USA based on the information provided by USDA [18] (banana, apple, grapes, watermelon, strawberry, orange, pear, tangerine, blueberry, lime, mango, pineapple, lemon, cantaloupe, and peach).

Food group 3 (Grains). The DGAs divide this group into whole and refined grains, suggesting the consumption of 3 ounces/day (84 g/day) of each. The WF was calculated as the average of the WFs of whole-wheat bread, brown rice, quinoa, and oats for the whole grains group and of white bread, white rice, pasta, and flaked cereals for the refined grains group. 

Food group 4 (Dairy). The DGAs suggest the consumption of 3 cups eq/day, without distinguishing between different dairy products. Therefore, we averaged the WF of the items listed in the examples (milk, yogurt, natural, and processed cheese), considering a different serving size for each food as indicated in the DGAs (1 cup eq: 244 g of milk, 43 g of natural cheese, and 57 g of processed cheese). Since, in the DGAs, there is no indication of the consumption of butter, it was not considered in the calculation of the WF of the diet.

Food group 5 (Protein Foods). In this group, the DGAs include (i) meat, poultry, and eggs; (ii) seafood; and (iii) nuts, seeds, and soy products. To calculate the WF of the first subgroup, the WFs of the recommended servings/week of beef, chicken, pork, and eggs were averaged, considering the serving size. For the seafood subgroup, the WF was calculated considering the water consumption per unit of production in aquaculture systems [16] and half of the suggested total consumption. In fact, the WF of wild fish is 0 L/kg, and it is estimated that 50% of the fish consumed comes from aquaculture [19]. For the nuts, seeds, and soy products subgroup, the WF of the suggested servings/week of tofu, shelled peanuts, pistachios, walnuts, hazelnuts, and almonds were averaged, considering serving sizes of 14 g of nuts and 45 g of tofu according to the examples of the guidelines. The type of nuts was chosen based on consumption habits in the USA [18]. 

Food group 6 (oils). According to USDA [18], the most consumed oils in the USA are soybean oil and canola oil. Consequently, the WFs of these oils were averaged.

Other foods. In a 2000 kcal/day dietary pattern, the DGAs indicate a limit of 240 kcal/day for added sugars, added refined starches, saturated fat, or alcohol. To allow for a comparison with the Italian diet, we included in this category sugar and jam, which were considered “indulgence” foods in the work of Bordoni A. [9]. The WF for sugar was the average of the WFs for beet and brown sugar, and the WF of jam was calculated based on 35% fruit and 40% sugar content, as in [9]. As the DGAs include alcoholic beverages in this food group, the WF was calculated as the average of the WF of sugar, jam, and alcohol—as the average between beer and wine—considering the quantity corresponding to 240 kcal.

### 2.3. Spain

The SDGs were used to elaborate a dietary pattern of 2000 kcal/day corresponding to a balanced diet for healthy Spanish adults. For this purpose, the recommendations on serving size and frequency of consumption of the different food groups were followed. As the SDGs do not give any indication about a daily energy intake but simply suggest a range of servings/day for each food group, two dietary patterns were elaborated, one considering the minimum and another the maximum of the suggested servings/day. Then, the two dietary patterns were averaged and the corresponding energy intake was calculated using the CREANUT food composition table [15]. Finally, the number of servings per week was proportionally adjusted to obtain a 2000 kcal/day dietary pattern, allowing the comparison to Italy and the USA. 

Food group 1 (Cereals and tubers). The WF was considered the average of the WF of bread, rice, pasta, breakfast cereals, and potatoes, which were the example foods reported in the guidelines. 

Food group 2 (Fruits). The WF of the group was considered the average of the WF of the most consumed fruits in Spain [20], i.e., orange, tangerine, peach, apple, pear, watermelon, melon, banana, and strawberries. 

Food group 3 (Vegetables). As for group 2, the WFs of the most consumed vegetables in Spain (tomato, pepper, zucchini, onion, lettuce, green beans, and spinach) were averaged and used for the calculation of the WF of the food group.

Food group 4 (Extra virgin olive oil—EVO). The WF of EVO was considered as the SDGs do not recommend the consumption of any other type of fat.

Food group 5 (Dairy). The guidelines suggest the consumption of 2–3 servings/day of dairy products, without discriminating between the foods. Therefore, the calculation was performed as reported for the USA. As Mekonnen et al. [12] do not distinguish between different cheeses, the same WF was used for cured and soft cheese, but different serving sizes were considered.

Food group 6 (Seafood). The WF of this food group was calculated as reported for the USA and Italy.

Food group 7 (Meat and eggs). The SDGs recommend consuming only white meat (3 servings/week). The consumption of red and processed meat is indicated as occasional and moderate, without any suggestion about the frequency of weekly consumption. Therefore, the WF of 0.5 serving/week of red/processed meat (bovine and pork) and the WF of 2.5 servings/week of chicken meat, the most consumed white meat according to the Spanish Ministry of Agriculture, Fisheries, and Food [20], were considered. The weight of 1 egg was considered the average of the range reported in the guidelines (53g–63g). 

Food group 8 (Pulses). As for the Italian diet, the WFs of dry beans, chickpeas, lentils, and peas were averaged. 

Food group 9 (Nuts). The WF was calculated as the average of shelled peanuts, pistachios, walnuts, hazelnuts, and almonds. 

Other foods. The SDGs do not provide information about the serving sizes or frequency of consumption of sugar or sweets but suggest occasional and moderate consumption. To obtain data comparable with the other considered countries, sugar and jam were considered with the same serving size and frequency of consumption used for Italy. The WF of 1.5 servings/day (average of maximum daily consumption for men and women suggested by the SDGs) of wine or beer (100 mL and 200 mL, respectively) was included in the calculation.

This study had a correlation research design, aiming to identify the relationships between variables without implying causation.

## 3. Results and Discussion

The total, green, blue, and grey WFs of the dietary patterns elaborated based on the IDGs, DGAs, and SDGs recommendations are reported in Table 1, Table 2 and Table 3, respectively.

The Italian dietary pattern showed the lowest total WF (2806 L per capita/day), with the American and Spanish patterns being 8% and 10.5% higher (3062 and 3137 L per capita/day, respectively). The results for Italy and Spain were in the range of the WF of European diets (2873–3792 L per capita/day) [21], while the total WF of the US dietary pattern was lower than that reported by Birney et al. [22]. This is justifiable because Birney et al. [22] considered food waste when analyzing WF_blue_, which was reported as 3560 L per capita/day. In this work, food waste was not considered and WF_blue_ was found to be 38% lower (2203 L per capita/day). 

**Figure 1:**
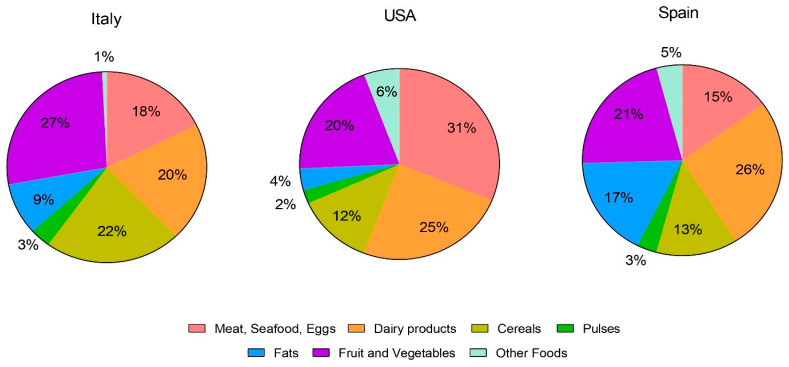
The contribution of different food groups to the total water footprint (WF) of the elaborated dietary patterns (Italy, the USA, and Spain).

Although the total WF was quite similar across the three dietary patterns, the food groups contributed differently (Figure 1).

In the United States, 56% of the total WF was attributable to animal products (meat, eggs, seafood, and dairy) versus 41% in Spain and 38% in Italy. Among animal-source foods, dairy products were the major contributors to the WFs of the Spanish and Italian diets (26% and 20%, respectively), while the largest contributors to the total WF of the US diet were meat, seafood, and eggs (32%), with dairy products accounting for 25%.

In recent years, the reduction in animal products in the diet has been supported by the scientific community not only for health reasons but also because it can contribute to the reduction in greenhouse gas emissions, the preservation of biodiversity, and the reduction in dietary WF [23]. Our results highlight that WF can vary depending on the animal products chosen while keeping a similar amount in weight. For example, although the IDG and the DGA suggest consuming a similar amount (3900 and 3750 g/week, respectively) of animal products (meat, eggs, seafood, and dairy), the recommendation for meat consumption is higher in the United States than in Italy (about 400 g/week more), where a higher consumption of dairy products is suggested. Replacing about 400 g of meat with the same amount of dairy would reduce the WF of the American diet by 3121 L per capita/week. As further confirmation of the importance of food choice, although the SDGs suggest a lower number of servings of dairy than IDGs (13 versus 24), the contribution of these products to the total WF was higher in the Spanish diet than in the Italian one (5724 versus 3943 L per capita/week). This difference is related to the fact that the IDGs specify that milk should cover 21 of the 24 portions/week suggested for the dairy food group, while the SDGs provide more general recommendations, indicating the number of servings without discriminating between different dairy products. Following the guidelines, the WF of dairy products in the Spanish diet was calculated as the average of milk and cheese—considering the different serving sizes—and this increased the WF of the food group.

This highlights that recommending a lower consumption of foods of animal origin to reduce the WF of the diet is simplistic and may not be effective if the type of product is not carefully evaluated. Although some animal products have similar nutritional characteristics, their WF could be very different. This applies to seafood, which has a lower WF/Kg than meat. The IDGs and SDGs suggest a similar weekly consumption of seafood (3.5 servings per week), while the US recommendation is lower (2.5 servings per week). Replacing one serving of poultry meat or red meat (125 g) with one serving of fish (125 g, with 50% from aquaculture, as considered in this work) could reduce the WF of the diet by 316 and 1965 L/week, respectively. On the one hand, this substitution should be encouraged in view of the inverse correlation between fish consumption and cardiovascular diseases [24]. On the other hand, excessive fish consumption could have consequences for both the environment and health. Indeed, it is known that fishing is the main factor in the modification of the marine ecosystem and the loss of marine biodiversity [25]. Furthermore, the consumption of some types of fish can lead to an increase in exposure to heavy metals [26]. This highlights the importance of increasing the sustainability of aquaculture and the need for a balanced approach when suggesting dietary changes. 

The contribution of plant foods to the WF was higher in Italy (61%) than in Spain (54%) and the USA (38%). In detail, in Italy, fruits and vegetables contributed 27%, cereals 22%, oils 9%, and pulses 3%, while the contributions of these food groups were 21%, 13%, 17%, and 3% in Spain and 20%, 12%, 4%, and 2% in the US. Food choice had a lower impact on the WF of plant foods than animal foods. Although different vegetables were considered based on the consumption habits of the three countries, the WF/Kg of these foods did not considerably differ (325, 400, and 327 L/Kg for Italy, the USA, and Spain, respectively). Moreover, considering different fruits slightly affected the WF/kg of the group (819, 690, and 618 L/Kg for Italy, the US, and Spain, respectively), with the higher WF/Kg in the Italian dietary pattern being justified by the fruits chosen (cherries, plums, and apricots), which have higher WFs than other fruits. 

Cereals contributed 22% in Italy, 13% in Spain, and 12% in the United States to the total WF, reflecting different dietary habits and preferences that were considered in the guidelines. In fact, these differences were mainly due to the quantity suggested for the food group (2717 g/week for Italy, 2120 g/week for the United States, and 2336 g/week for Spain) since the average WF/kg of the selected foods was similar. The guidelines suggest predominant consumption of minimally processed grains, and it should be considered that the choice of refined grains or highly processed foods could easily lead to significant changes in the WF. For example, the difference between bread and more highly processed analogs (e.g., crackers) is 500 L/kg. 

In all dietary patterns, pulses were the food group with the lower impact on the total WF (2–3%), except the “other food” group in the Italian diet. This reflected the low recommended quantity of legumes per week, which ranged from 110 to 160 g of dried legumes. The substantial nutritional [27] and environmental advantages of pulses are equally compelling [28]. By acting as natural carbon sinks and establishing a symbiotic relationship with nitrogen-fixing bacteria, legume crops contribute to reducing greenhouse gas emissions and increasing soil fertility [29]. Other advantages of pulses are their resistance to environmental threats and culinary flexibility [29]. Adding one serving of dry pulses (50 g)/week and subtracting the number of meats providing the same protein concentration would decrease the WF of 570 L/week, thus confirming the importance of promoting the consumption of pulses.

**Figure 2:**
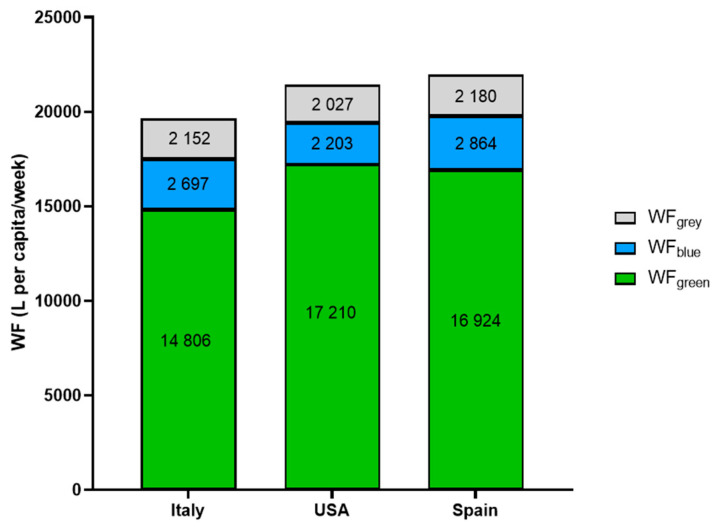
Breakdown of the total WF into the green, blue, and grey components.

The distribution of the total WF between WF_green_, WF_blue_, and WF_grey_ was similar in the three dietary patterns, and green water was always the predominant consumption (75%, 80%, and 77% of the total WF for Italy, the USA, and Spain, respectively) (Figure 2). The higher WF_green_ of the American and Spanish diets compared to the Italian one is justified by the higher recommended consumption of animal products, especially meat, and extra virgin olive oil, respectively.

The contributions of WF_blue_ to the total WF of the diet were 14%, 10%, and 13% in the Italian, US, and Spanish diets, respectively. WF_blue_ is linked to precipitation [30], as the more it rains, the less irrigation is needed. As climate change will have an increasing impact on precipitation patterns, WF_blue_ may change considerably. Across all dietary patterns, fruit was the main contributor to WF_blue_ (Italy with 41%, the United States with 34%, and Spain with 32%), followed by cereals for Italy (27%), dairy products for the United States (21%), and EVO for Spain (22%). The contribution of fruit to WF_blue_ was not proportional to the consumption recommendation (4074 g for Italy, 3175 g for the US, and 4350 g for Spain), further confirming that the type of fruit has an impact. 

One of the future challenges to improve the water requirements of food production will be the identification of crops or cultivars that have low water requirements. Although the selection of crops and a careful choice of foods can lead to a significant reduction in WF, it must be considered that there are foods that, due to their nutritional value and their traditional nature, can hardly be reduced or replaced. EVO is culturally part of the eating habits of Mediterranean countries, and the preventive effects of its consumption are documented [31,32]. It is difficult to recommend switching to oils with WF lower than EVO, even if its total WF is similar to that of red meat and its WF_blue_ is more than 4 times higher than that of meat. However, even in the Mediterranean area, the suggested consumption of EVO varies between countries, and the SDGs suggest the consumption of 260 g/week of EVO while the IDGs suggest the consumption of 140 g of vegetable oils in general. These different recommendations determined a significant difference (+2421 L/week) in the total WF between Spain and Italy. It would therefore be important to carefully evaluate the preventive effects of EVO in order to suggest consumption that is effective but not too high so as not to impact the WF. 

The contribution of WF_grey_ was similar across the three dietary patterns (11% for Italy and 10% for the US and Spain). Although nuts had the highest WF_grey_/kg, the low recommended intake of these foods (60–95 g/week) dwarfed their contribution to dietary WF_grey_, which was mostly related to fruit, meat, and dairy products. Reducing WF_grey_ is particularly challenging because it is linked to water safety and quality, which are critical to public and environmental health. Therefore, since water quality standards cannot change, strategies to reduce WF_grey_ must focus on improving agricultural practices and water management systems. This may include minimizing the use of agrochemicals and adopting agricultural methods that reduce water pollution [33]. 

This study has limitations. First, to set dietary patterns, we only considered foods specifically included in the guidelines and for which the serving size and frequency of consumption were specified. Although this was an easily reproducible method that allowed the comparison of dietary recommendations from different countries, the development of menus that include coffee, tea, alcohol, spices, sugar, and highly processed foods could lead to a more realistic WF assessment. Indeed, Blas et al. [34] calculated the WF considering two weekly menus based on guidelines but including some “indulgent foods” and highlighted higher values than those reported in this study (5276 L per capita/day for Spain and 5632 L per capita/day for the United States). Indeed, the inclusion of spices and herbs (cinnamon, garlic, pepper, coriander, cumin, vanilla, oregano, rosemary, and bay leaves, 290 g/week in total) and other “indulgence foods” (chocolate, coffee, and mustard, 220 g/week in total) as reported by Blas et al. [34], resulted in an increase of 843 L/day of total WF, which would be further increased by the inclusion of more processed foods (e.g., tomato paste versus tomato: 855 L/Kg versus 214 L/kg). However, the foods contributing most to the WF of dietary patterns were the same in [34] and in our study.

Another limitation is related to the different structure of the guidelines. Foods are grouped differently; for example, the DGAs include soy products in the “nut” group, while Italy and Spain consider them part of the “pulses” group. Additionally, some guidelines specify the frequency of consumption of different items in a food group, while others simply indicate a general frequency of consumption for the entire food group. This can lead to significant differences in the WF of the group, as reported above for dairy products.

## 4. Conclusions

The nutritional requirements of healthy adults are universal; however, the specific combinations of foods used to meet these needs can vary widely, reflecting the cultural practices, dietary habits, and traditional food systems of different regions. Therefore, different dietary patterns can be equally sustainable from a nutritional point of view, and therefore provide all the necessary nutrients, but have a different impact on environmental sustainability. Indeed, the results of this study highlight that diets elaborated on the suggestions of national dietary guidelines have a different WF, underlining that the type of foods chosen within the food groups plays a fundamental role. 

One of the key findings emerging from the data reported is related to the relationship between the environmental impact and consumption of animal products. Although attention to their environmental sustainability has increased in recent years, it seems clear that simply reducing their consumption may not be sufficient to obtain environmental benefits and that careful consideration of the specific type of product is essential. Furthermore, all the possible environmental consequences induced by a change in dietary patterns must be considered. For example, replacing red meat with fish could reduce the total WF but could have consequences related to overfishing. The relationship between the consumption of animal products and environmental sustainability is a delicate issue, which also depends on the farming system of animal products. Caution is therefore needed in balancing nutritional, environmental, and social needs when trying to optimize the consumption of animal products. It seems easier to optimize the choice of plant-based foods by selecting crops and varieties with a lower WF per kilogram, considering that even culturally significant products, such as EVO, require a balanced approach.

In addition, our study highlights the lack of specific data for the assessment of WF. Until they are available, it will not be possible to fully assess the impact of new cultivation methods, differences in climate and irrigation, and agricultural ecological diversity in different regions. Equally, it will be important to produce data that take into account the production phase and food waste and that can highlight the importance of good practices in the agri-food industry for the reduction in WF. This appears particularly important in the future because the impact of climate change on precipitation could lead to greater demand for blue water, which is difficult to obtain when water availability is a problem.

In conclusion, further studies are needed to foster dialogue between nutritionists, environmentalists, and agri-food professionals in order to ensure a healthy and balanced approach that takes into account the impact on water resources.

## Figures and Tables

**Table 1:** Water footprint of the 2000 kcal Italian dietary pattern based on the IDGs. (^a^) Converted considering the percentage of the edible part, when needed.

Food Category	Serving Size (g of Edible Portion)	Suggested Consumption (Serving/Week)	Suggested Consumption (g/Week) ^a^	WF (L/kg or L/L)				WF of the Suggested Consumption (L/Week)			
				Green	Blue	Grey	Total	Green	Blue	Grey	Total
**Food group 1—meat (terrestrial and aquatic animals) and eggs**											
Red meat	**100**	1	113	9661	505	537	10,702	1092	57	61	1209
White meat	100	2	286	3545	313	467	4325	1014	90	134	1237
Fish/seafood	150	2	428	815	90	83	987	349	38	36	422
Processed fish	50	1	50	815	90	83	987	41	4	4	49
Eggs	50	3	172.5	2592	244	429	3265	447	42	74	563
**Food group 2 (Milk and dairy)**											
Milk/yoghurt	125	21	2625	863	86	72	1020	2265	226	189	2678
Soft cheese	100	2	200	4264	439	357	5060	853	88	71	1012
Hard cheese	50	1	50	4264	439	357	5060	213	22	18	253
**Food group 3 (Cereals and potatoes)**											
Bread	50	24.5	1225	1124	301	183	1608	1377	369	224	1970
Bread analogue	80	10.5	840	1562	322	223	2108	1312	270	188	1770
Pasta, rice, barley	30	1	30	1126	322	113	1608	34	10	3	48
Sweet bakery (croissants)	50	1	50	2843	812	284	4062	142	41	14	203
Sweet bakery (biscuits)	30	1	30	2140	611	214	3057	64	18	6	92
Breakfast cereals	30	2	60	1998	245	173	2416	120	15	10	145
Potatoes	200	2	482	191	33	63	287	92	16	30	138
**Food group 4 (Pulses)**											
Dry pulses	50	3	150	3174	218	879	4271	476	33	132	641
**Food group 5 (Fats)**											
Vegetable oil	10	14	140	7994	1324	313	9631	1119	185	44	1348
Butter	10	7	70	4965	465	393	5553	348	33	28	389
**Food groups 6 and 7 (Fruit and vegetables)**											
Fresh fruit	150	21	4074	540	171	108	819	2200	695	441	3337
Nuts (shelled)	30	2	60	5761	3253	1231	10,245	346	195	74	615
Leafy vegetables	200	10.5	2919	239	69	94	402	698	201	274	1173
Other vegetables	80	7	742	147	21	104	271	109	16	77	201
**Indulgence food**											
Sugar	5	10.5	52.5	860	327	137	1324	45	17	7	70
Jam	20	2	40	1277	416	321	2018	51	17	13	81
**Total**								14,806	2697	2152	19,645

IDGs = Italian Dietary Guidelines; WF = water footprint.

**Table 2:** Water footprint of the 2000kcal American dietary pattern based on the DGAs. (^a^) Converted considering the percentage of the edible part, when needed.

Food Category	Serving Size (g of Edible Portion)	Suggested Consumption (Serving/Week)	Suggested Consumption (g/Week) ^a^	WF (L/kg or L/L)				WF of the Suggested Consumption (L/Week)			
				Green	Blue	Grey	Total	Green	Blue	Grey	Total
**Food group 1 (Vegetables and Pulses)**											
Green vegetables	60	1.5	133	170	60	96	325	23	6	12	41
Red and orange vegetables	145	5.5	916	201	32	68	301	184	30	62	276
Starchy vegetables	161	5	944	757	49	113	918	744	43	97	884
Other vegetables	112	4	563	292	76	206	574	164	43	116	323
Dry Pulses	74	1.5	111	3174	218	879	4271	352	24	98	474
**Food group 2 (Fruit)**											
Fruit	161	14	3175	458	141	92	690	1372	424	266	2062
**Food group 3 (Cereals)**											
Whole grains	28	21	588	2059	254	150	2463	1211	149	88	1448
Refined grains	28	21	588	1531	351	211	2093	900	206	124	1230
**Food group 4 (Protein food)**											
Dairy	115	21	2408	3130	321	262	3713	4459	454	373	5284
Meat, poultry, eggs	34	26	964	6365	392	492	7248	5550	354	465	6369
Seafood	28	8	320	815	90	83	987	261	29	27	316
Nuts, seed, soy products	19	5	96	5200	2724	1034	8958	426	193	73	692
**Food groups 6 (Oils)**											
Oils	27	7	189	3603	288	355	4246	681	54	67	802
Limit on calories for other use (240 kcal/day)	260	7	1823	749	224	152	1127	882	193	161	1238
**Total**								17,210	2203	2027	21,440

DGAs = Dietary Guidelines for Americans; WF = water footprint.

**Table 3:** Water footprint of the 2000kcal Spanish dietary pattern based on the SDGs. (^a^) Converted considering the percentage of the edible part, when needed.

Food Category	Serving Size (g of Edible Portion)	Suggested Consumption (Serving/Week)	Suggested Consumption (g/Week) ^a^	WF (L/kg or L/L)				WF of the Suggested Consumption (L/Week)			
				Green	Blue	Grey	Total	Green	Blue	Grey	Total
**Food group 1 (Cereals and potatoes)**											
Cereals and potatoes	79	27	2336	1204	252	166	1621	2118	458	325	2902
**Food group 2 (Fruit)**											
Fruit	175	19	4350	412	112	95	618	1793	486	412	2691
**Food group 3 (Vegetables)**											
Vegetables	200	13	3061	189	38	103	327	579	118	316	1003
**Food group 4 (Oils)**											
Extra Virgin Olive Oil	14	19	261	11,826	2388	217	14,431	3089	624	57	3769
**Food group 5 (Dairy)**											
Dairy	125	13	1664	2564	263	215	3040	4829	493	404	5724
**Food group 6 (Seafood)**											
Seafood	125	3	476	815	90	83	987	387	43	39	469
**Food group 7 (Meat and eggs)**											
White meat	125	2	317	3545	313	467	4325	1124	99	148	1371
Red meat	125	0.4	53	14,414	550	451	15,415	762	29	24	815
Eggs	58	3	255	503	47	83	633	503	47	83	634
**Food group 8 (Pulses)**											
Dry Pulses	70	2	160	3174	218	879	4271	508	35	141	683
**Food group 9 (Nuts)**											
Nuts	25	4	95	5761	3253	1231	10,245	548	310	117	975
**Indulgence food**											
Sugar	5	10.5	1668	858	327	137	1324	45	17	7	69
Jam	20	2	40	1277	416	321	2018	51	17	13	81
Wine/beer	150	10.5	1575	431	77	76	584	585	89	93	769
**Total**								16,924	2864	2180	21,958

SDGs = Spanish Dietary Guidelines; WF = water footprint.

## Data Availability

The data that support the findings of this study are openly available in Zenodo at https://doi.org/10.5281/zenodo.14276159.
